# JOSD2 regulates PKM2 nuclear translocation and reduces acute myeloid leukemia progression

**DOI:** 10.1186/s40164-022-00295-w

**Published:** 2022-07-14

**Authors:** Hu Lei, Li Yang, Yingying Wang, Zhihui Zou, Meng Liu, Hanzhang Xu, Yingli Wu

**Affiliations:** 1 Hongqiao International Institute of Medicine, Shanghai Tongren Hospital/Faculty of Basic Medicine, Key Laboratory of Cell Differentiation and Apoptosis of the Chinese Ministry of Education, Shanghai Jiao Tong University School of Medicine, Shanghai, 200025 China; 2 Research Units of Stress and Tumor (2019RU043), Chinese Academy of Medical Sciences, School of Medicine, Shanghai Jiao Tong University, Shanghai, 200025 China

**Keywords:** JOSD2, PKM2, Acute myeloid leukemia, Nuclear localization

## Abstract

Pyruvate kinase M2 (PKM2) plays an important role in the metabolism and proliferation of leukemia cells. Here, we show that deubiquitinase JOSD2, a novel tumor suppressor, blocks PKM2 nuclear localization by reducing its K433 acetylation in acute myeloid leukemia (AML). Firstly, we show that JOSD2 is significantly down-regulated in primary AML cells. Reconstitute of JOSD2 in AML cells significantly inhibit cell viability and induce cell apoptosis. Next, PKM2 is identified as a novel interaction protein of JOSD2 by mass spectrometry, co- immunoprecipitation and co-immunofluorescence in HL60 cells. However, JOSD2 does not affect PKM2 protein stability. We then found out that JOSD2 inhibits nuclear localization of PKM2 by reducing its K433 acetylation modification, accompanied by decreased downstream gene expression through non-glycolytic functions. Finally, JOSD2 decreases AML progression in vivo. Taken together, we propose that JOSD2 blocks PKM2 nuclear localization and reduces AML progression.

**Supplementary Information:**

The online version contains supplementary material available at 10.1186/s40164-022-00295-w.

To the Editor,

Acute myeloid leukemia (AML) is a hematological malignancy characterized by the accumulation of immature myeloid cells with distinct molecular genetic characteristics. Although targeted therapies and immunotherapies, such as BCL2 inhibitors and FLT3 inhibitors, have achieved some clinical efficacy, the overall survival rate for AML remains low. Understanding the pathogenesis of AML is important for the development of new targeted drugs to improve the treatment of AML.

The biological functions of Machado-Joseph deubiquitinases (MJDs) in cancer are gradually being revealed [1, 2]. MJDs consist of four members, namely, Ataxin-3, Ataxin-3L, JOSD1 and JOSD2. JOSD1 and JOSD2 have received less attention before, but they have been identified to be associated with cancer progression recently. JOSD2 preferentially recognizes substrates containing K11, K48, and K63 linkages, suggesting a possible role in maintaining protein quality control [3]. JOSD2 has been shown to promote non-small cell lung cancer (NSCLC) cell proliferation by stabilizing metabolic enzymes aldolase A and phosphofructokinase-1, suggesting that JOSD2 is a positive regulator of glucose metabolism [4]. In NSCLC, JOSD2 also deubiquitinates and stabilizes phosphoglycerate dehydrogenase, a key enzyme that drives the first committed step in de novo serine biosynthesis [5]. JOSD2 also stabilizes YAP/TAZ to promote cholangiocarcinoma progression [6]. However, the function of JOSD2 in AML is still unknown. In this study, we reveal the role of JOSD2 in the pathogenesis of AML.

Using real-time RT-PCR, we found that JOSD2 and JOSD1 were expressed at lower levels in different types of primary AML cells compared with normal peripheral blood mononuclear cells (PBMCs) (Fig. 1a). The details of AML patients were provided in Additional file 1: Table S1. The protein levels of JOSD2 and JOSD1 were also abnormally reduced in primary AML cells compared to normal PBMCs (Fig. 1b). Then, we further detected the expression of JOSD2 in normal PBMCs, primary AML PBMCs and HL60 cells by immunofluorescence, and the results showed that the expression of JOSD2 in AML cells was also inhibited (Additional file 1: Fig. S1a). We also demonstrated that JOSD2 expression was decreased in AML cell lines at transcription and protein levels (Additional file 1: Fig. S1b, c). Next, we overexpressed JOSD2 and JOSD1 in AML cell lines, respectively, and the results showed that JOSD2 overexpression significantly inhibited cell viability compared with JOSD1 (Fig. 1c). JOSD2 overexpression significantly inhibited the expression of Bcl2 and Mcl-1, but induces cleavage of caspase-3 (except HL60 cells) and increased the proportion of apoptotic cells in AML cells (Fig. 1d and Additional file 1: Fig. S2a). Meanwhile, we found that overexpression of JOSD2 did not significantly induce AML cells differentiation by CD11b staining (Additional file 1: Fig. S2b). In order to further find out the mechanism of JOSD2, mass spectrometry analysis was performed in JOSD2-overexpressed HL60 cells after immunoprecipitation with Flag antibody. The results showed that pyruvate kinase M2 (PKM2) interacts with JOSD2 (Fig. 1e, f). Furthermore, we further demonstrated that JOSD2 and PKM2 co-located in cytoplasm by immunofluorescence (Fig. 1g). However, JOSD2 did not affect PKM2 protein level (Fig. 1h), but immunofluorescence showed that JOSD2 overexpression could significantly inhibit PKM2 nuclear localization in HL60 and primary AML cells (Fig. 1i, j and Additional file 1: Fig. S3a). Cytoplasmic and nuclear isolation experiments further demonstrated that PKM2 nuclear localization was regulated by JOSD2 (Fig. 1k).

**Fig. 1:**
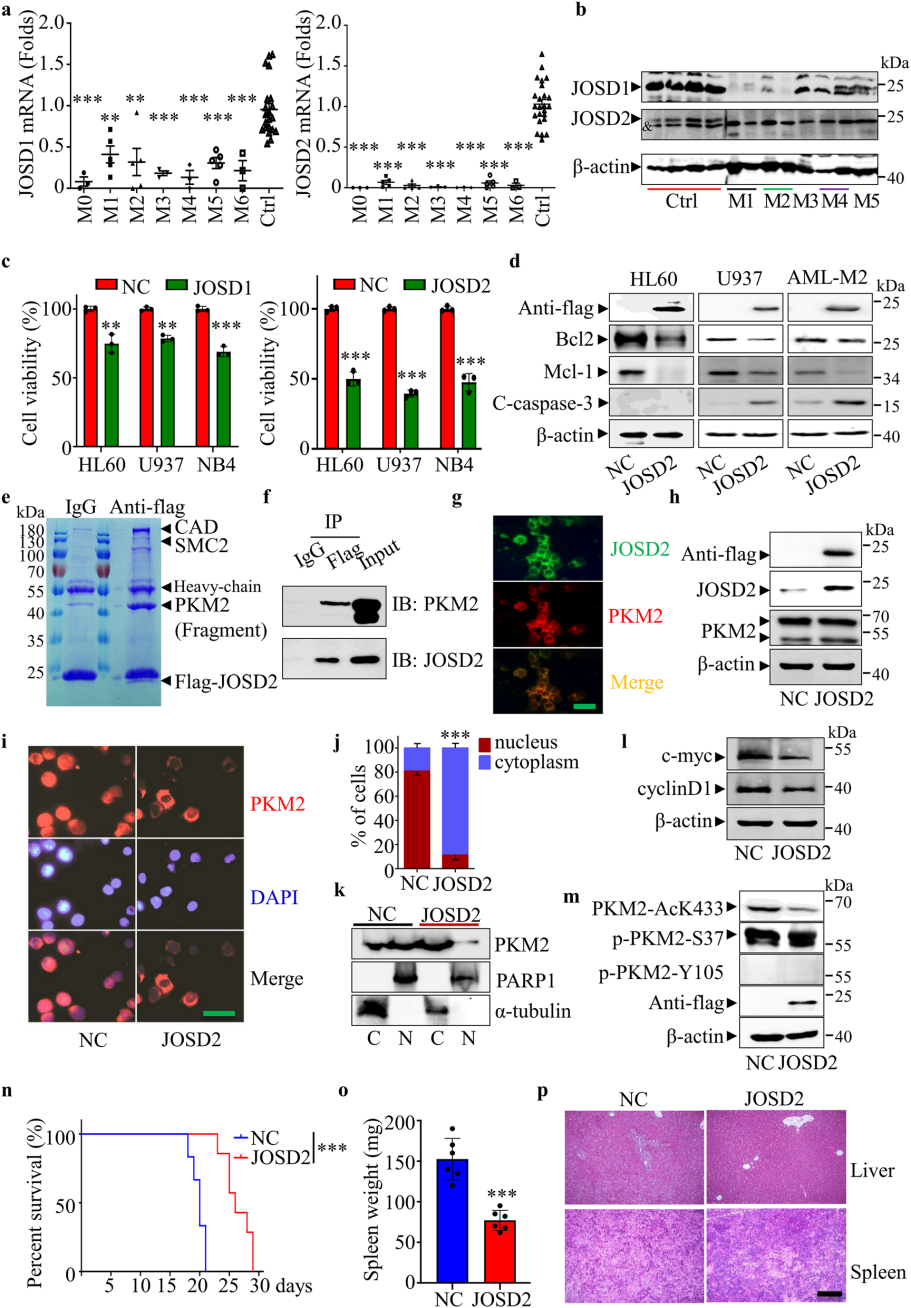
**a** mRNA levels of JOSD1 and JOSD2 in peripheral blood mononuclear cells (PBMCs) from primary AML patients compared with normal PBMCs (M0 = 3, M1 = 5, M2 = 5, M3 = 3, M4 = 3, M5 = 5, M6 = 3, normal n = 24). Data are presented as mean ± SD, and were analyzed by using One-way ANOVA. **P < 0.01, ***P < 0.001. **b** Western blot analysis of JOSD1 and JOSD2 protein expression in PBMCs from health donors and primary AML patients. ^&^represents a band of nonspecific signals. **c** Cell viability of HL60, U937 and NB4 leukemia cells after JOSD1 and JOSD2 overexpression was measured by CCK-8 assay. Data are presented as mean ± SD, and were analyzed by using the 2-tailed Student t test. **P < 0.01, ***P < 0.001. **d** Western blot analysis of protein expression as shown in the figure after restoring JOSD2 expression in HL60, U937 and primary AML cells. **e** Immunopurification and mass spectrometric analysis of JOSD2-interacting proteins in JOSD2-overexpressed HL60 cells. **f** Co-immunoprecipitation assay showing the interaction between JOSD2 and PKM2 in JOSD2-overexpressed HL60 cells. **g** JOSD2 and PKM2 were co-located in the cytoplasm of HL60 cells overexpressing JOSD2. **h** Western blot analysis of PKM2 expression after restoring JOSD2 expression in HL60 cells. **i** Immunofluorescence assay shows that JOSD2 blocks PKM2 nuclear localization in HL60 cells. Scale bar, 20 μm. **j** The number of PKM2cells in cytoplasm or nucleus was counted. **k** The localization of PKM2 in HL60 cells with low JOSD2 expression and high JOSD2 expression was detected by nuclear cytoplasmic separation. Data are presented as mean ± SD, and were analyzed by using the 2-tailed Student t test. ***P < 0.001. **l** Western blot analysis of c-myc and cyclin D1 expression after restoring JOSD2 expression in HL60 cells. **m** The effects of JOSD2 on K433 acetylation and phosphorylation of S37 and Y105 of PKM2 were detected by westernblot. **n** JOSD2 or NC transfected HL60 cells were injected into B-NDG mice (n = 6 in NC grounp, n = 7 in JOSD2 grounp) through the tail vein. The survival was analyzed by Mantel-Cox-log-rank test. ***P < 0.001. **o** The spleen of leukemia-mice was weighed at day 16. Data are presented as mean ± SD, and were analyzed by using the 2-tailed Student t test. ***P < 0.001. **p** H&E staining of liver and spleen from mice bearing leukemia at day 16. Scale bar, 200 μm

The nuclear-localized PKM2 plays a regulatory role in gene expression with other transcription factors. For example, PKM2/β-catenin and PKM2/Nrf2 reveal the expression of different genes, such as cyclinD1 and c-myc, which are related to lipid biosynthesis and glutathione biosynthesis et al. [7]. JOSD2 overexpression reduces cyclinD1 and c-myc expression at the transcriptional and protein levels (Fig. 1l and Additional file 1: Fig. S3b). Serine/Threonine phosphorylation or acetylation of PKM2 at specific sites results in nuclear localization of PKM2 [8]. We found that JOSD2 overexpression inhibited the K433 acetylation of PKM2, but had no effect on the phosphorylation of S37 and Y105 (Fig. 1m). Finally, we demonstrated that JOSD2 inhibits AML progression in vivo. Compared with the control group, JOSD2-overexpressed HL60 cells prolong the survival of B-NDG mice with smaller spleen, less tissue damage and less leukemia cell infiltration. (Fig. 1n–p).

In conclusion, our work identifies JOSD2 is a novel tumor suppressor and demonstrates that PKM2 is a novel JOSD2 interacting protein in AML. In addition, we reveal that JOSD2 blocks nuclear localization of PKM2 by decreasing its K433 acetylation in AML.

## Supplementary Information

**Additional file 1: Table S1.** The information of AML patients. **Figure S1.** Expression of JOSD2 in AML. a Immunofluorescence analysis of JOSD2 protein expression in PBMCs from health donors, primary AML patients (M2) and AML cell lines. Scale bar, 20 μm. b mRNA levels of JOSD2 in AML cell lines compared with normal peripheral blood mononuclear cells. c Western blot analysis of JOSD2 protein expression in bone marrow mononuclear cells from health donors and AML cell lines. Data are presented as mean ± SD, and were analyzed by using One-way ANOVA. ***P < 0.001. **Figure S2.** Changes of apoptosis and differentiation after overexpression of JOSD2 in AML cells. **a** Cell apoptosis was determined by flow cytometric analysis of Annexin V and PI staining. **b** Cell differentiation was determined by flow cytometric analysis of side-scatter profiles (SSC) and the expression of CD11b. Data are presented as mean ± SD, and were analyzed by using the 2-tailed Student t test. **P < 0.01, ***P < 0.001. **Figure S3.** JOSD2 blocks PKM2 nuclear localization and inhibited the expression of the related genes cyclin D1 and c-myc. **a** Immunofluorescence assay shows that JOSD2 blocks PKM2 nuclear localization in primary AML (M5) cells. Scale bar, 20 μm. **b** JOSD2 expression inhibited the transcription levels of cyclin D1 and c-myc, the downstream target genes of PKM2 nuclear localization. Data are presented as mean ± SD, and were analyzed by using the 2-tailed Student t test. ***P < 0.001.

## Data Availability

Not applicable.

## Abbreviations

AML: Acute myeloid leukemia
MJDs: Machado-Joseph deubiquitinases
NSCLC: Non-small cell lung cancer
PKM2: Pyruvate kinase M2
PBMCs: Peripheral blood mononuclear cells
