# Supplementary material for: JOSD2 regulates PKM2 nuclear translocation and reduces acute myeloid leukemia progression
Source: Exp Hematol Oncol. 2022 Jul 14;11:42. doi: 10.1186/s40164-022-00295-w (PMC9281007; doi:10.1186/s40164-022-00295-w)
Supplement: Supplementary file 1 — Additional file 1: Table S1. The information of AML patients. Figure S1. Expression of JOSD2 in AML. a Immunofluorescence analysis of JOSD2 protein expression in PBMCs from health donors, primary AML patients (M2) and AML cell lines. Scale bar, 20 μm. b mRNA levels of JOSD2 in AML cell lines compared with normal peripheral blood mononuclear cells. c Western blot analysis of JOSD2 protein expression in bone marrow mononuclear cells from health donors and AML cell lines. Data are presented as mean ± SD, and were analyzed by using One-way ANOVA. ***P < 0.001. Figure S2. Changes of apoptosis and differentiation after overexpression of JOSD2 in AML cells. a Cell apoptosis was determined by flow cytometric analysis of Annexin V and PI staining. b Cell differentiation was determined by flow cytometric analysis of side-scatter profiles (SSC) and the expression of CD11b. Data are presented as mean ± SD, and were analyzed by using the 2-tailed Student t test. **P < 0.01, ***P < 0.001. Figure S3. JOSD2 blocks PKM2 nuclear localization and inhibited the expression of the related genes cyclin D1 and c-myc. a Immunofluorescence assay shows that JOSD2 blocks PKM2 nuclear localization in primary AML (M5) cells. Scale bar, 20 μm. b JOSD2 expression inhibited the transcription levels of cyclin D1 and c-myc, the downstream target genes of PKM2 nuclear localization. Data are presented as mean ± SD, and were analyzed by using the 2-tailed Student t test. ***P < 0.001. [file 40164_2022_295_MOESM1_ESM.pdf]

Additional File for:

JOSD2 regulates PKM2 nuclear translocation and reduces acute myeloid leukemia progression

Hu Lei[1†*], Li Yang[1†], Yingying Wang[1], Zhihui Zou[1], Meng Liu[1], Hanzhang Xu[1*], Yingli Wu[1,2*]

[1]Hongqiao International Institute of Medicine, Shanghai Tongren Hospital/Faculty of Basic Medicine, Key Laboratory of Cell Differentiation and Apoptosis of the Chinese Ministry of Education, Shanghai Jiao Tong University School of Medicine, Shanghai 200025, China

[2] Shanghai Jiao Tong University, Research Units of Stress and Tumor (2019RU043), Chinese Academy of Medical Sciences, Sch Med, Shanghai 200025, China

†These authors contributed equally to this work

*Correspondence

Yingli Wu. E-mail: wuyingli@shsmu.edu.cn

Hanzhang Xu. E-mail: xuhanzhang@shsmu.edu.cn

Hu Lei. E-mail: hulei@shsmu.edu.cn

MATERIALS AND METHODS

Reagents and antibodies

PKM2 (4053), cleaced-caspase-3 (9664), cytochrome C (4280), p62 (8025), c-myc (9402), cyclin D1 (2922) and phospho-PKM2 (Tyr105) (3827) antibody were purchased from Cell Signaling Technology. BCL2 (sc-7382) and MCL1 (sc-12756) antibody were purchased from Santa Cruz Biotechnology. Antibody specific for α-tubulin (11224-1-AP), flag tag (66008-2-Ig) and β-actin (60008-1-Ig) were purchased from Proteintech. JOSD1 (ab118221) antibody was purchased from Abcam. JOSD2 (OAAB00616) antibody was purchased from Aviva Systems Biology. Phospho-PKM2 (Ser37) (GTX133886) antibody was purchased from GeneTex. PKM2-Ac433 antibody was provided by professor Qunying Lei. Flag-bead (M20018) was purchased from Abmart. Nuclear protein and cytoplasmic protein extraction kit (P0027) was purchased from Beyotime.

Cell culture

HL60, NB4 and U937 were maintained in RPMI-1640 (Gibco, 21875109) supplemented with 10% heat-inactivated fetal bovine serum (Gemini, 900-108), penicillin (50 U/ml)/ streptomycin (50 μg/ml) (Sangon Biotech, E607011). All cells were cultured at 37 ℃ in a humidified atmosphere of 5% CO_2.

Quantitative RT-PCR

Total RNA was extracted with Trizol reagent (Invitrogen) and reverse transcription into cDNA by Reverse Transcriptase kit (Takara). SYBR (Roche) and Applied Biosystems Step One Plus™ detection system (ABI 7900) were used for real-time quantitative PCR. Specific primers are shown as follows: JOSD1 F: GTG GAT TGG AGG CGA GAG, R: AGC AGG AGT TCA CAG TTC TT; JOSD2 F: TGA GAT CTG CAA GAG GTT, R: ATC ACA TTG ACA TCA TAG TTG; Cyclin D1 F: GCG GAG GAG AAC AAA CAG, R: GCG GTA GTA GGA CAG GAA, C-myc F: GCG ACT CTG AGG AGG AA, R: TGC GTA GTT GTG CTG ATG; GAPDH F: CTT AGC ACC CCT GGC CAA G, R: TGG TCA TGA GTC CTT CCA CG.

Immunoprecipitation and immunoblot analysis

Flag-JOSD2 overexpressed HL60 cells were prepared with lysis buffer (Beyotime, P0013) and incubated overnight with Anti-flag beads at 4℃ overnight. Wash with PBST buffer for three times and separate with SDS-PAGE. These proteins are transferred to nitrocellulose membranes and detected with appropriate antibodies.

Immunofluorescence

The cultured cells were fixed to a slide by a cytospin and fixed with 0.3% Triton X-100. After permeabilization with 100% cold methanol and blocking with 2% (w/v) bovine serum albumin, the cells were incubated JOSD2 antibody (1:100 dilution) or/and PKM2 antibody (1:100 dilution) overnight at 4 °C. Then, the slides were stained with the secondary antibody and DAPI. The fluorescence signal of the cells was detected by confocal microscopy (Nikon, A1R-si).

B-NDG mice model

B-NDG mice, NOD-SCID IL-2 receptor gamma null mice, 6-week-old were obtained from Jiangsu Biocytogen Co., Ltd (Nantong, China). HL60-NC and HL60-JOSD2 cells (5×10^6) were injected into the tail vein of six or seven mice per group. When the mice developed humpback and/or hind limb paralysis, they were killed and their spleens were measured. The survival of mice from the tail vein model is represented with a Kaplan-Meier survival plot.

Statistical analysis

All data are present as mean ±SD. One-way ANOVA was used for analyzing significant difference of multiple groups, Student's *t*-test was used to determine significances between two groups. All statistical tests were performed using the GraphPad Prism 8, P values<0.05 were considered to be statistically significant.

Table 1 The information of AML patients

AML type	Gender	Age	WBC(×10^9)	Blast（%）	Genetic abnormality
M0	female	43	75.39	28	NPM1
	male	55	135.9	34	FLT3-ITD
	female	62	85.23	38	NA
M1	female	16	6.48	62	FLT3-ITD
	male	12	6.78	52	CEBPA
	female	64	7.96	62	NA
	female	46	8.31	70	CEBPA, ASXL1
	female	22	4.38	63.5	CEBPA
M2	male	26	15.4	57.5	AML1-ETO+, c-kit
	male	32	6.1	27	AML1-ETO+, c-kit
	male	62	8.8	59	ASXL, CEBPA, TET2
	male	49	28.33	45	NA
	male	24	26.54	43	RUNX1, IDH2
M3	female	20	9.26	58	PML-RARα
	female	14	6.83	63	PML-RARα
	female	25	6.69	81	PML-RARα
M4	female	24	31.1	35	DNMT3A
	female	58	28.9	28	DNMT3A, IDH2, NRAS
	male	50	49.6	52.5	DNMT3A, IDH2, TET2
M5	male	42	23.83	39	NA
	male	35	3.84	45.5	NPM1
	male	60	1.1	51	TET2
	female	40	67.69	89	FLT3-ITD
	female	23	6.06	23	TET2
M6	female	33	2.13	56	NA
	female	18	8.23	58	NPM1
	male	44	5.63	49	FLT3-ITD

NA: not available.

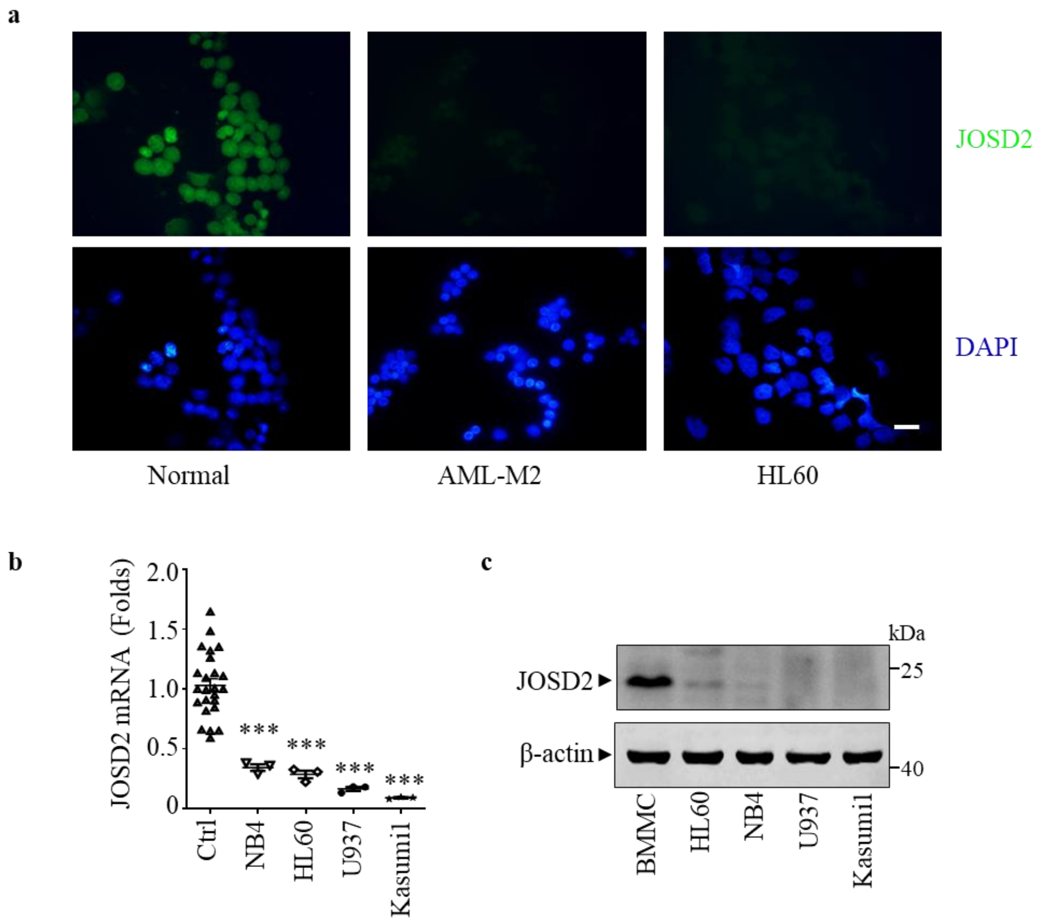

Figure 1 Expression of JOSD2 in AML. a Immunofluorescence analysis of JOSD2 protein expression in PBMCs from health donors, primary AML patients (M2) and AML cell lines. Scale bar, 20 μm. **b** mRNA levels of JOSD2 in AML cell lines compared with normal peripheral blood mononuclear cells. **c** Western blot analysis of JOSD2 protein expression in bone marrow mononuclear cells from health donors and AML cell lines. Data are presented as mean ± SD, and were analyzed by using One-way ANOVA. ****P*<0.001

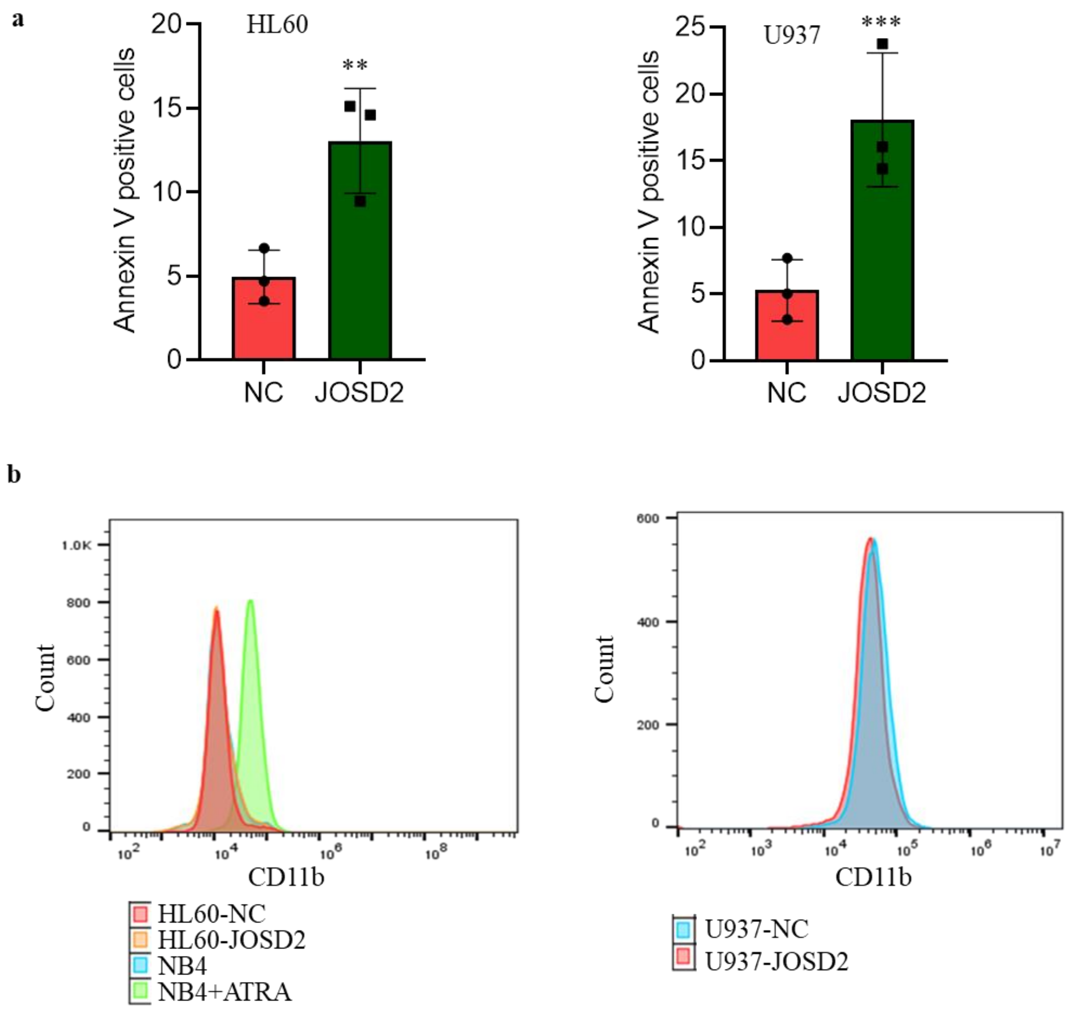

Figure 2 Changes of apoptosis and differentiation after overexpression of JOSD2 in AML cells. a Cell apoptosis was determined by flow cytometric analysis of Annexin V and PI staining. **b** Cell differentiation was determined by flow cytometric analysis of side-scatter profiles (SSC) and the expression of CD11b. Data are presented as mean±SD, and were analyzed by using the 2-tailed Student t test. **P<0.01, ***P<0.001.

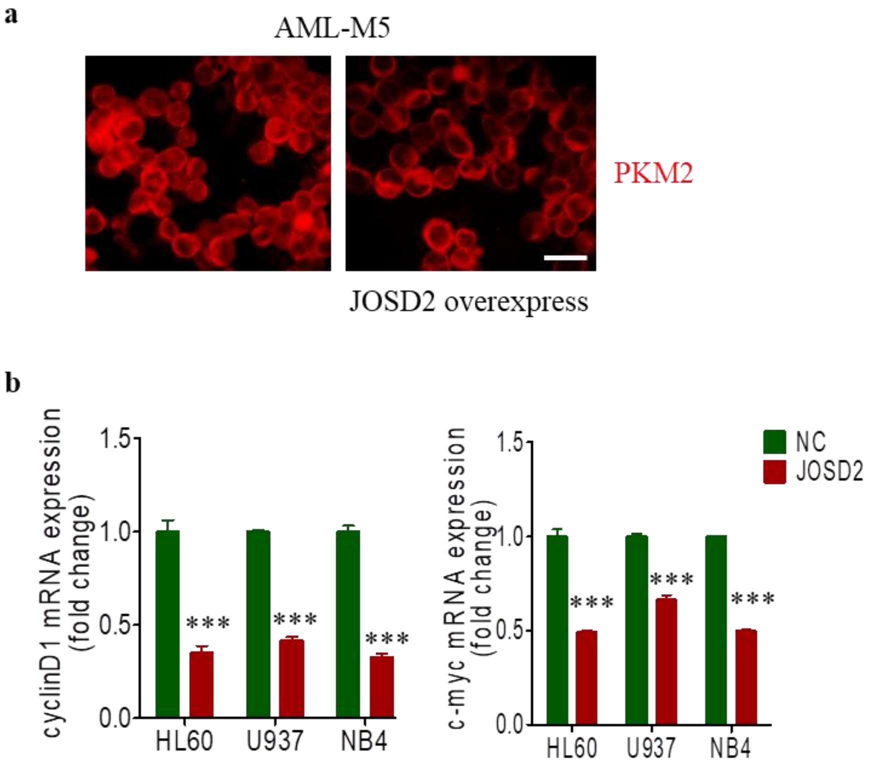

Figure 3 JOSD2 blocks PKM2 nuclear localization and inhibited the expression of the related genes cyclin D1 and c-myc. a Immunofluorescence assay shows that JOSD2 blocks PKM2 nuclear localization in primary AML (M5) cells. Scale bar, 20 μm**. b** JOSD2 expression inhibited the transcription levels of cyclin D1 and c-myc, the downstream target genes of PKM2 nuclear localization. Data are presented as mean ± SD, and were analyzed by using the 2-tailed Student t test. ****P*<0.001.
